# Detection of extremely low concentration waterborne pathogen using a multiplexing self-referencing SERS microfluidic biosensor

**DOI:** 10.1186/s13036-017-0051-x

**Published:** 2017-02-14

**Authors:** Chao Wang, Foram Madiyar, Chenxu Yu, Jun Li

**Affiliations:** 1 Agricultural and Biosystems Engineering Department, Iowa State University, Ames, IA 50011 USA; 2 Chemistry department, Kansas State University, Manhattan, KS 66506 USA

## Abstract

**Background:**

It is challenging to achieve ultrasensitive and selective detection of waterborne pathogens at extremely low levels (i.e., single cell/mL) using conventional methods. Even with molecular methods such as ELISA or PCR, multi-enrichment steps are needed which are labor and cost intensive. In this study, we incorporated nano-dielectrophoretic microfluidic device with Surface enhanced Raman scattering (SERS) technique to build a novel portable biosensor for easy detection and characterization of *Escherichia coli O157:H7* at high sensitivity level (single cell/mL).

**Results:**

A multiplexing dual recognition SERS scheme was developed to achieve one-step target detection without the need to separate target-bound probes from unbound ones. With three different SERS-tagged molecular probes targeting different epitopes of the same pathogen being deployed simultaneously, detection of pathogen targets was achieved at single cell level with sub-species specificity that has not been reported before in single-step pathogen detection.

**Conclusion:**

The self-referencing protocol implements with a Nano-dielectrophoretic microfluidic device potentially can become an easy-to-use, field-deployable spectroscopic sensor for onsite detection of pathogenic microorganisms.

## Background

Pathogen detection and identification is of the utmost importance for medicine, food safety, public health and security, and water and environmental quality control [1]. The World Health Organization (WHO) identified that contaminated water serves as a mechanism to transmit communicable diseases such as diarrhea, cholera, dysentery, typhoid and guinea worm infection. Except for poor water, sanitation and hygiene services (WASH) conditions in communities and institutional settings, slow detection strategies have also been exacerbating the spread of those infectious diseases. Timing is extremely important in pathogen detection and the delay or inaccurate diagnosis of the pathogenic infection is always the primary cause of mortality or serious illness. Traditional and standard pathogen detection methods rely on off-line laboratory procedures (consist of multiple cultural enrichment steps, isolation of bacterial colonies, identification) and may take up to 8 days to yield an answer [2]. This slow process clearly can’t provide a sufficient protection from exposure to water borne pathogens within public drinking water. Outside traditional culturing, many methods have been developed to promote the detection efficacy, such as polymerase chain reaction (PCR), enzyme-linked immunosorbent assay (ELISA), and surface plasmon resonance (SPR) sensors [3–6]. These techniques provide high selectivity and reliability; however, they usually require intensive sample preparation and special equipment and trained users [7]. Furthermore, in reality, the competitor organisms in water samples can cross-react with detection systems, rendering false-positive results, or can grow to levels that will mask target organisms. Hence, there is a compelling need for the development of easy-to-use biosensors that could give highly sensitive and reliable detection results, and even allow on-site field monitoring [8].

Surface-enhanced Raman scattering (SERS), as a label-free/non-destructive optical technique, has been widely used in pathogen discrimination [9–12]. The distinct “fingerprinting” Raman spectra of microorganisms can be enhanced at rough noble metal nanostructures’ surfaces, which is essentially important in pathogen detection since discrimination of different bacterial species and strains is difficult. Recently, various nanostructures with different surface features have been employed to amplify the enhancement of SERS signals in bacterial identifications at cellular and molecular levels. However, it is still a challenge to obtain repeatable and reproducible SERS spectroscopic results at complicated experimental conditions. The degree of metallic nanoparticles aggregation, the different size of metal colloids, and the inhomogeneous distributions of nanoparticles on cells all affect the SERS signal reproducibility. To overcome those limitations, specific antibodies and Raman tags molecules are introduced into nanostructures to probe the target biomolecules and produce a high-specific and reproducible SERS signals [13–15]. However, the simultaneous presence of nanoparticles, SERS reporters, and biological samples generates highly overlapping and complex spectra which make it difficult to identify the target bacteria. Therefore, it is necessary to integrate statistical analysis techniques into bacterial SERS discrimination for data mining [14, 16–20].

**Fig. 1:**
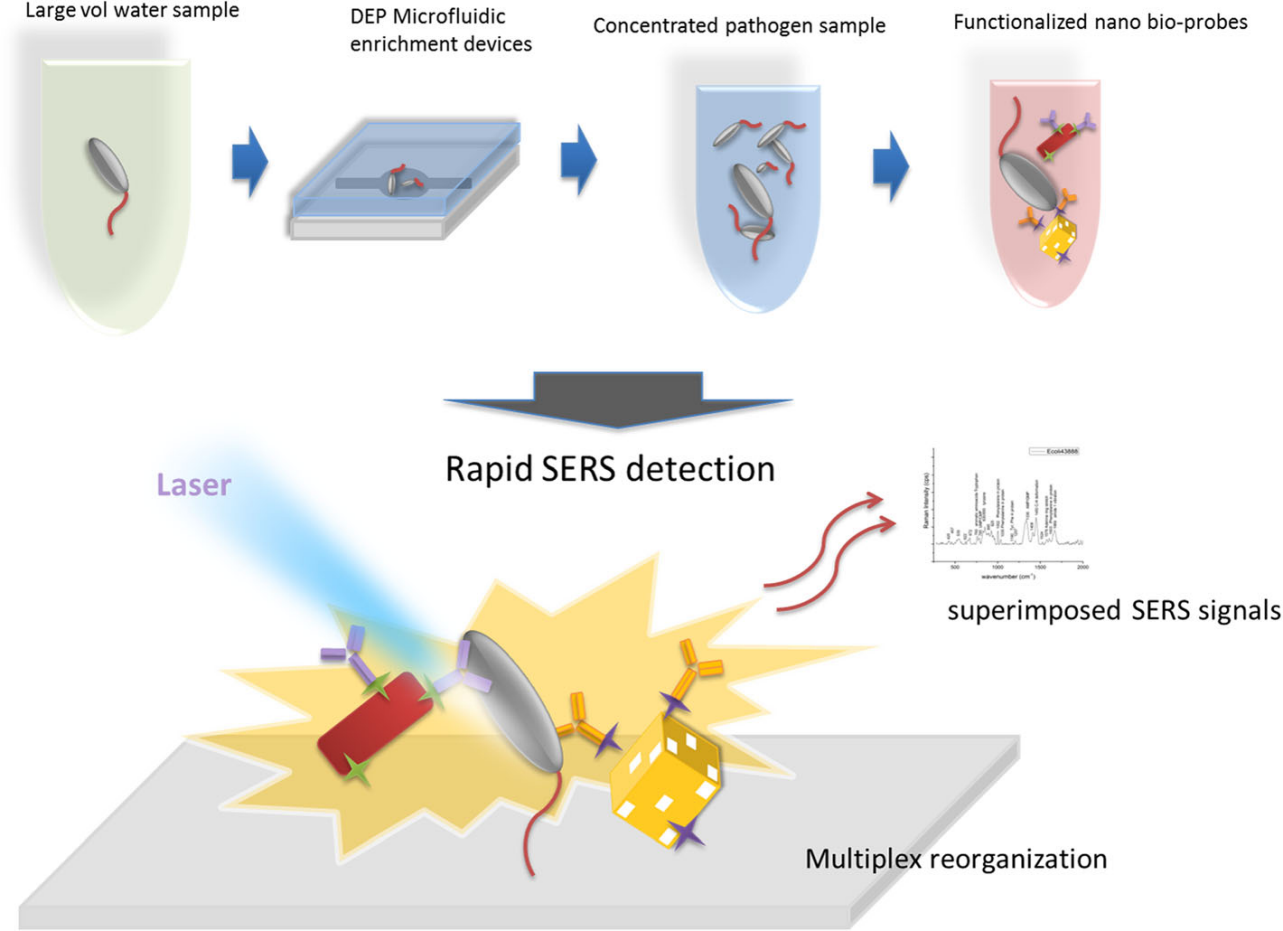
Schematic routine describing the rapid enrichment step using microfluidic device and detection step using the multiplex self-referencing SERS strategy

Herein, we developed the concept of self-referencing mechanism that utilized SERS molecular probes to achieve target bacteria detection in one single step with high reliability brought by a novel multiplex targeting scheme, and integrated multivariate statistical analysis methods to simplify the superimposed SERS spectra for rapid and accurate diagnostics of water samples. To further improve the limit of detection (LOD) in the pathogenic bacteria detection strategy, and to facilitate possible deployment as on-site detection apparatus, a bacterial concentration mechanism based on nano-dielectrophoretic (Nano-DEP) enrichment was integrated with the SERS signal acquisition/analysis to yield a microfluidic sample preparation platform (Fig. 1). Although in recent years, quite a few reports on DEP-based microfluidic biosensors have been published [21–24], including a few with SERS as the detection mechanism [25–28], almost all of the relevant past work used microbial samples with high concentrations (>10^6^ CFU/mL) for DEP operations. In this study, we investigated samples with microbial concentration at 1–10 CFU/mL, which is more relevant in terms of potential practical applications, such as monitoring pathogens in drinking water.

## Methods

### Chemical and biological materials

Hexadecyltrimethylammoniumbromide (CTAB, ≥99%); Gold(III) chloride trihydrate (HAuCl_4_.3H_2_O, 99.9 + %); Sodium borohydride (NaBH_4_, ≥99%); Silver nitrate (AgNO_3_, ≥99%); L-Ascorbic acid (AA, ≥99.0%); 4-Aminothiophenol (4-ATP, 97%); 3-Amino-1,2,4-triazole-5-thiol (ATT, 95%); Phosphate-buffered saline (PBS), 10× concentrate. Ethylene glycol (EG, 99%), sodium sulfide (Na_2_S, 99%); Polyvinylpyrrolidone (PVP, 99%); 3-Mercaptopropionic acid (≥99%). All reagents are purchased from Sigma-Aldrich. *E.coli O157: H7* (No. 43888) and *E.coli K12* (29425) frozen-dried strains were purchased from ATCC (Manassas, VA, USA). Anti-*E.coli* antibodies were purchased from Abcam (Cambridge, MA, USA). 18.2 MΩ.cm E-pure water is used for all regents’ preparation.

### Bacterial sample preparation for Raman spectroscopic analysis

Different bacterial strains were cultured in petri dishes of 60 mm × 15 mm that have a layer of agar-based Luria Broth medium. After 18 h 37°C incubation, the desired bacteria colonies were inoculated in liquid Luria Broth medium for liquid culture. After 18 h incubation at 37°C, bacterial solution was transferred to 15 mL centrifuge tubes and concentrated under 3000 RPM speed for 3 min. After removing the supernatant, the dense pellets of bacteria were obtained for subsequent Raman identification tests or series dilution.

### Functionalization Gold nanorods (GNRs) with 4-ATP and ATT and antibodies

GNRs were synthesized following the standard protocol in literatures [29]. 3 mL of 10 mM 4-ATP and ATT (pH=2) was added into 24 mL GNR-CTAB with LSPR OD (optical density) =6. The mixture solution was kept in disposable scintillation vials at 60°C oil bath with 180 rpm stirring speed for around 19 h. Then, functionalized GNRs solution was washed twice by centrifugation (6000×g for 10 min) with 20mM CTAB and pH=4 pure water. Finally suspend the products in 0.25 mL water. For antibody conjugation, 0.75 μg anti-*E.coli O157:H7* mouse monoclonal antibodies (P3C6, ab75244) were incubated with 500 μL GNR-4ATP (OD=11.2); 0.75 μg another anti-*E.coli O157:H7* mouse monoclonal antibodies (3011, ab20976) were incubated with 500 μL GNR-ATT.

### Functionalization of Cage with 3-MPA and antibodies

Gold cages were synthesized following the standard protocol in literatures [30]. The 3-MPA-gold nanocages were prepared by ligand-exchange reaction between 3-MPA and PVP stabilized gold nanocages. The cages solution were diluted to 100 mL with OD=1.0. Ligand-exchange reaction was performed at room temperature by mixing the prepared cage solution with a 100 μL of aqueous solution of 20 mM 3-MPA under shaking treatment. The mixed solution was treated overnight under the room temperature. After centrifuging, the supernatant were removed. The pellet was washed with pure water for 1 time. For antibody conjugation, 0.75 μg anti-*E.coli O157:H7* rabbit polyclonal antibodies (HRP ab68450 from Abcam) were incubated with 500 μL Cage-3MPA.

### SERS measurement

DXR Raman microscope (Thermo Scientific, Waltham, MA, USA) was used for Raman spectra acquisition with 780 nm excitation at 10 mW, 10× objective, and 25 μm slit. The laser exposure time was 5 s and spectral resolution was 2.4–4.4 cm^−1^. Different batches of nanoprobes were used for each mixed sample to test the reproducibility of the SERS measurement. Several droplets of sample solutions were placed on gold-coated microscope slide, and multiple SERS spectra were obtained from different positions on each droplet. The OMNIC™ suite (Thermo Scientific, Waltham, MA, USA) was used for data processing. The focusing point in the colloidal state liquid sample is randomly selected for all collection in order to obtain a big and random database to fulfill the requirement in the following statistical analysis.

### Nano-DEP microfluidic device operation

By using the microfluidic device, cell enrichment could be achieved in a continuous sample preparation step. Two different *E.coli* strains were mixed: *E.coli O157:H7* (pathogenic) and *E.coli K12* (non-pathogenic) and used to test the efficiency of the microfluidic device. The mixture was used so that the specificity of the self-referencing mechanism in the following SERS measurement can be investigated. Two *E. coli* strains were diluted to extremely low concentrations and mixed together uniformly. 1 mL of the mixed cell suspension at 10^0^ CFU/mL was passed through the microfluidic device. At a flow rate of 1*μ*L per min, it took about 17 h for the 1 mL sample to be processed. The concentrated samples then were collected.

### Statistical analysis

The spectra were firstly baseline-corrected, smoothed and area normalized. An iterative polynomial background removal algorithm was implemented to remove background fluorescence from the Raman spectral data [31].

Principal components analysis (PCA) is a common statistical technique that is used to reduce the number of dimensions of data with a minimum loss of information [19]. The goal of PCA is to determine the data patterns and underlying factors that cause the similarities and differences of the original data without any prior knowledge. Those factors are orthogonal basis and called principal components (PCs). For each PC score, the influence (weight) of the original spectral data is found in its corresponding loading profile. In this study, PCA was performed using MatLab (Mathworks, Inc., Natick, MA).

## Result and discussion

### Multiple bioconjugated gold nanoparticles (AuNPs) as SERS nanoprobes for bacterial identification at 10 CFU/mL

**Fig. 2:**
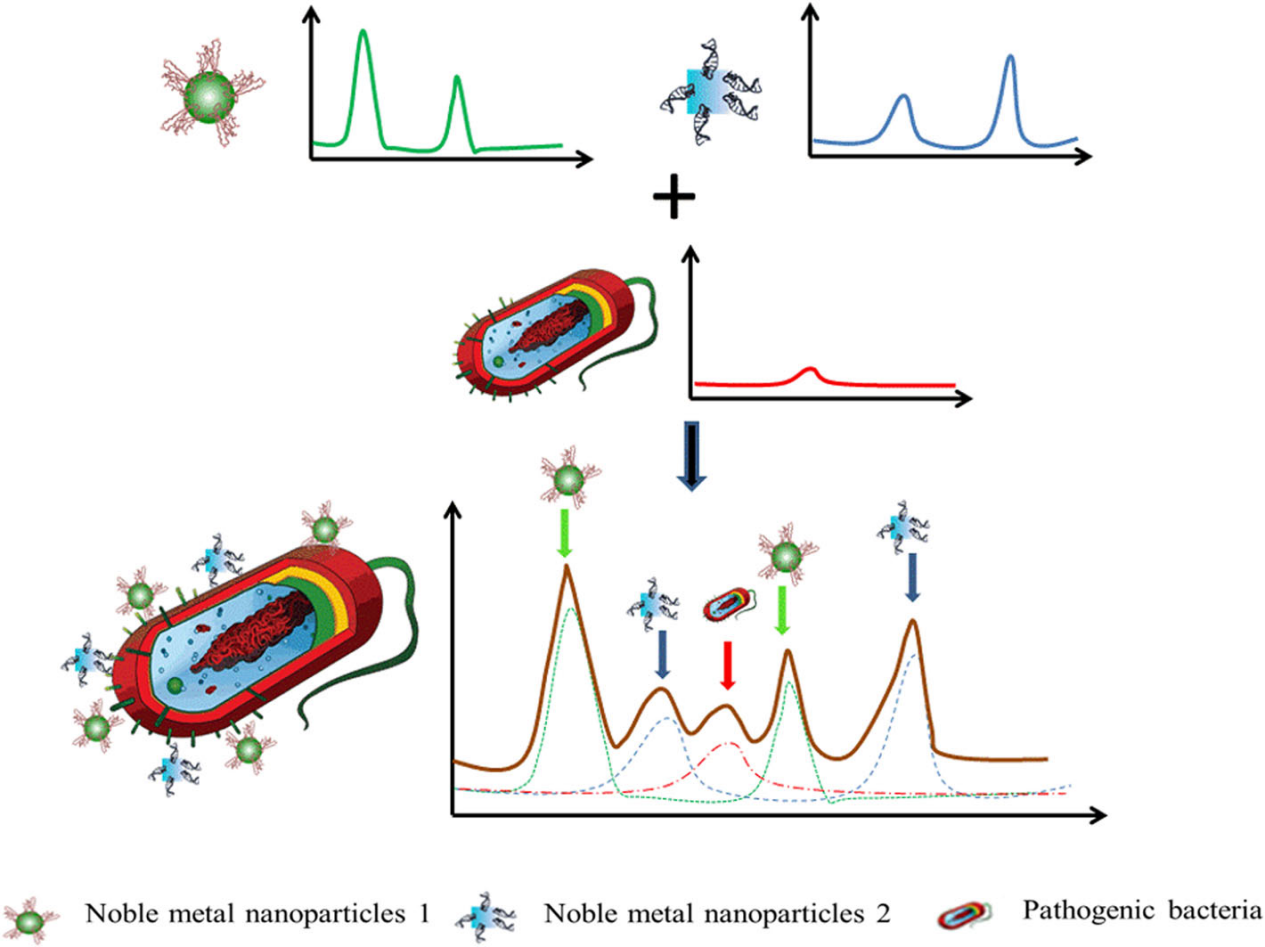
Schematic of Multiplex self-referencing pathogen recognition using SERS molecular probes

The mechanism of the self-referencing scheme of pathogen detection using two probes targeting two epitopes of the same pathogen is shown in Fig. 2. Only specific binding of nanoprobes to targets will yield detectable dual SERS signal (i.e., probe+target signals), non-specific binding or no-binding will not yield dual signals in this scheme, because only the specially designed SERS probes, made from functionalized anisotropic nanoparticles, can generate enough enhancement to the bacterial-originated signal to make it SERS-detectable [23]. However, in the single epitope mechanism we reported earlier [32], the chance of probes not binding properly to the targets was still quite high. To further improve the detection specificity and signal enhancement by reducing the miss-binding, we developed a three-epitope detection scheme to avoid the antibody-antigen binding failures in one-epitope setup. Briefly, three different types SERS probes with monoclonal antibodies binding to three different epitopes on the same pathogen cell were deployed against a pathogen target, each of the probe-target binding events become more distinctive and specific due to the appearance of different SERS tag signals with various enhanced signature peaks. Three different Raman tag molecules (4-Aminothiophenol, 4-ATP; 3-Amino-1,2,4-triazole-5-thiol, ATT; and 3-Mercaptopropionic acid, 3-MPA) are selected as probe reporters for nanoparticle functionalization, because of their large Raman cross section, and favorable chemistry for antibody conjugation. The three selected Raman tag molecules have weak signals in the microbial spectral range so that the microbial signals would not be overwhelmed by the enhanced Raman tags peaks in SERS spectral analysis.

**Fig. 3:**
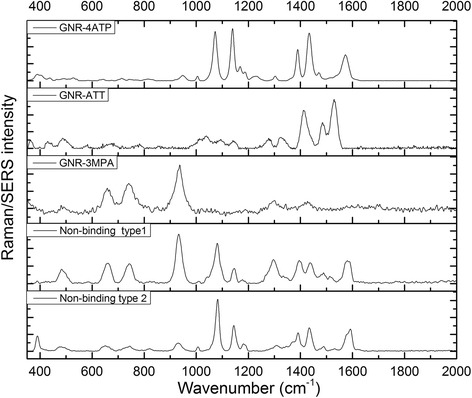
SERS spectra of three nanoprobes. Upper three spectra: nanorod-4ATP-monoclonal antibody; nanorod-ATT-monoclonal antibody; nanocage-3MPA-polyclonal antibody. Down two spectra: SERS spectra of two subtypes of non-binding

Two anisotropic AuNPs, nanorods and nanocages, were employed as SERS enhancers because anisotropic particles show stronger electromagnetic enhancement compared to isotropic structures [33]. After covalently coupling the three tags molecules on nanostructures, respectively (4-ATP and ATT conjugated to nanorods, and 3-MPA conjugated on nanocages), three different anti-E.coli O157: H7 antibodies were conjugated to the tags molecules by diazo bonding reaction and EDC/NHS bonding reaction. The SERS spectra of three nanoprobes are shown in Fig. 3. By comparing the Raman intensities of these three probes, the enhancement factors from diazo bonding conjugation (4-ATP-antibody and ATT-antibody) were larger than EDC/NHS bonding (3-MPA-antibody). The nanorod-4ATP-antibody showing the highest enhancement factor.

In our self-referencing scheme, the SERS signatures of the target bacteria were observed superimposed with the SERS signals of the Raman tags. The assessment through the dual signals (superimposed target and tag Raman signatures) supported a specific recognition of the targets in a single step with no washing/separation needed. However, the complex nature of the SERS imparts the implementation of the self-referencing scheme with a lot of variations. Dual signals could only be observed when the conjugated bacterial cell wall components fall into the hotspot regions of the nanoprobes. Hot spots are highly localized regions of intense local field enhancement believed to be caused by local surface plasmon resonances. In practice, the variation of nanoprobe conjugation location and density on the surface of the bacterial cells was very high. Therefore, the enhanced-type spectra were not expected in every single measurement. Among all the spectra collected, most were non-enhanced Raman spectra (data not shown) in which the significantly enhanced Raman fingerprint signatures were not identifiable from both Raman tag and bacteria; and 20% of the acquired spectra were considered as SERS spectra. Among the SERS spectra, two types could be identified: one was non-binding (probe alone) type in which only the featured peaks from three Raman tag molecules could be found, indicating that the probes were not bound to bacterial cells; the other was binding (dual signal) type in which both Raman tags’ peaks and bacterial peaks were significantly enhanced. The multiplex scheme we employed to detect multiple epitopes further added to the complication of the analysis. As shown in Fig. 3, even the non-binding spectra do not always show identical characteristics: in some measurements the three probe signals were not all detectable at the same intensity level. The randomly occurrence of hot spots is to blame for this inconsistency.

**Fig. 4:**
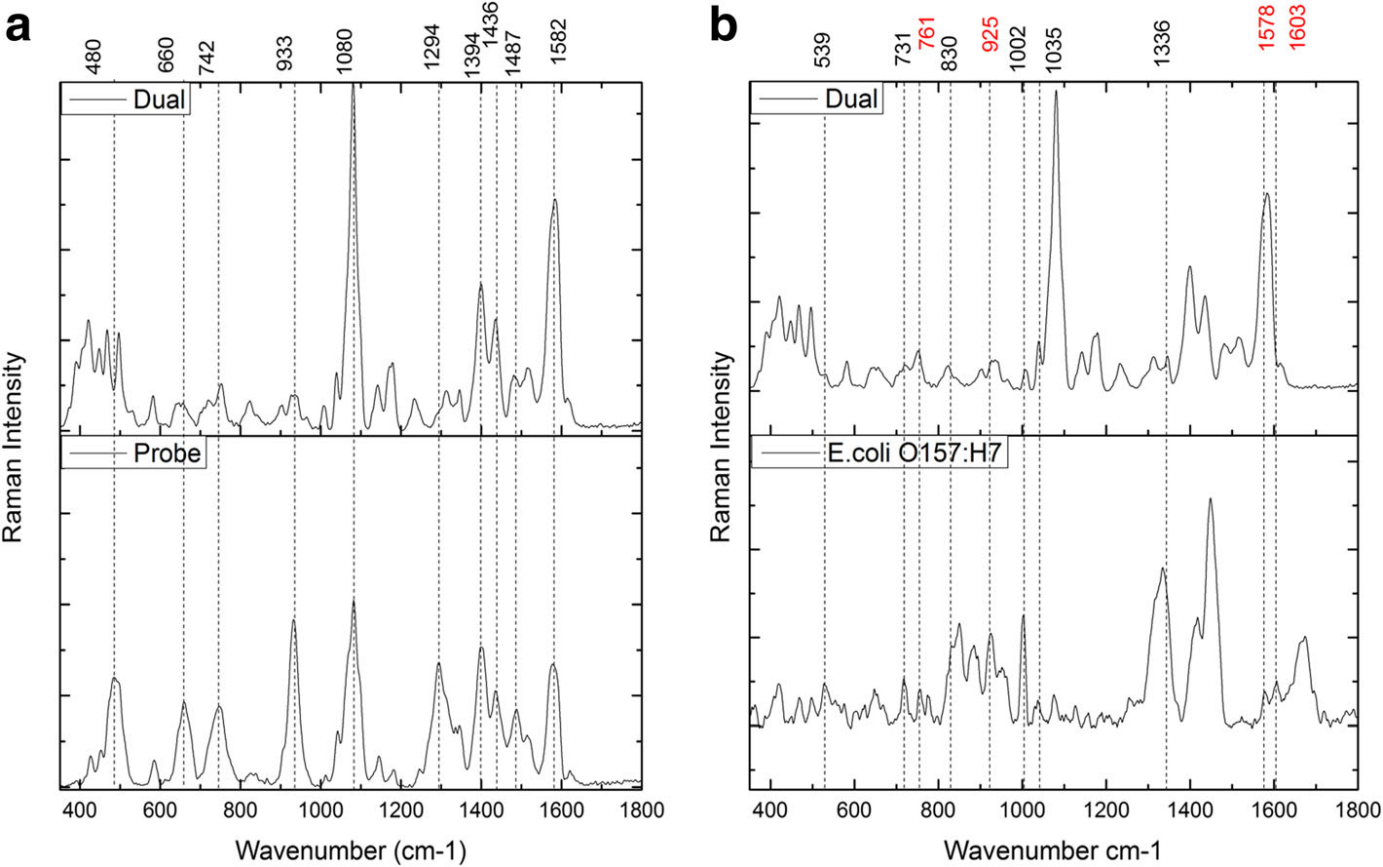
SERS spectral results of bacterial samples using Nano-DEP microfluidic device. **a** SERS spectra and peak assignment of non-binding (probe signal) type and binding (dual signal) type (the marked peaks are the featured Raman peaks from three Raman tag molecules); (**b**) SERS spectra and peak assignment of binding type and normal Raman spectra of pure & high-concentration *E.coli O157: H7* bacteria (the marked peaks are the Raman signatures from target pathogen, the red color marked ones cannot be clearly identified in the dual-type spectra)

The average probe (i.e., non-binding) and dual (i.e., binding) types SERS spectra are shown in Fig. 4a. The peaks from Raman tags are assigned at the top of this figure. Besides the Raman tags SERS peaks, some weaker but identifiable peaks could be found in the microbial information rich region (500–1000 cm^−1^). The peak assignment for bacterial components (bacteria at 10^1^ CFU/mL) in dual SERS spectra are shown in Fig. 4b. The pure and high-concentration *E.coli O157:H7* is also exhibited in this figure as reference (see Table 1 for peak assignment). Although the dual spectra of the sample can be easily differentiated from the probe-alone spectra of the control, the reproducibility of the SERS-based self-referencing analysis was still not ideal. The variations could be due to the nature of the SERS process, where even the same analyte (i.e., chemically heterogeneous bacterium) cannot be expected to repeatedly satisfy all of the criteria for SERS, namely, orientation, and presence within the range of the enhanced local optical field [34]. Furthermore, the surface enhancement of nanostructures is highly distance dependent and the electromagnetic field decays exponentially away from the nanoparticle’s surface. Hence, the Raman tag molecules, as the nearest conjugated layer, exhibit the highest Raman peaks intensity. On contrast, the outside layer bacterial cell wall components show weaker peak intensity. Additionally, some of the important microbial components (marked as red color wavenumber) peaks could not be clearly identified due to the overlapping with the Raman tags featured signals. All these complexity makes the self-referencing scheme not very consistent. To assure consistent and reproducible detection is to be achieved, statistical analysis needs to be used.

**Table 1:** Band assignment of *E.coli O157:H7* featured peaks shown in multiple self-referencing SERS measurement

Wavenumber (cm^−1^)	Band assignment
1660	Amide 1 vibration
1603	Phenylalanine in protein
1578	Adenine ring stretching
1336	γ(NH_2_) adenine, polyadenine, Phenylalanine in protein
1035	Phenylalanine in protein
1002	Phenylalanine in protein
925	CN stretching
830/850	Tyrosine
761	Aromatic aminoacids/Tryptophan
731	adenine, glycosidic ring mode, DNA
539	S-S bond stretching

### Multivariate statistical analysis for rapid discrimination and classification of target bacteria

**Fig. 5:**
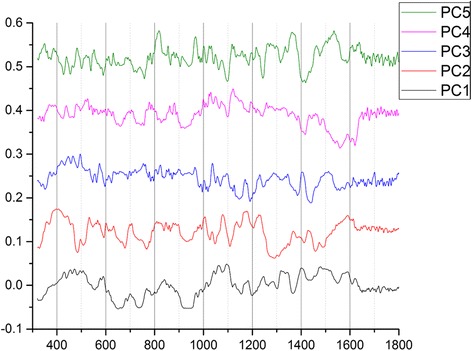
The first 5 principal component loadings of the PCA performed on the SERS spectra acquired from multiplex antibodies functionalized Nanoprobes conjugating with *E.coli O157: H7* bacteria sample

**Fig. 6:**
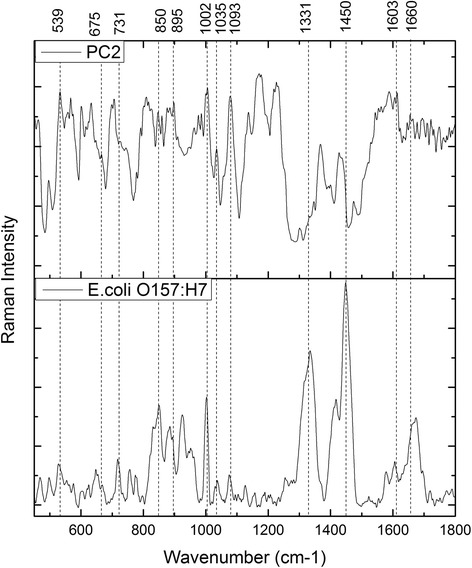
The peak identification of PC2 spectral loading and the Raman spectrum of pure & high-concentration Bacterial target cells

To qualify the spectral differences responsible for discrimination, the first 5 PC loadings (see Fig. 5) were compared to find the most representative loading spectra that could be originated from bacterial targets. It is considered that the second PC loading spectrum showed the highest correlation with the bacterial Raman spectra. The spectral contribution from the second PC loading is assigned in Fig. 6, in comparison to the Raman spectra of the high-concentration *E.coli O157:H7*. The 2^nd^ PC loading shows Raman signature bands of target pathogen matching at 731, 850, 1002, 1035, 1093, 1331, 1603, 1660 cm^−1^ (see Table 1 for reference). This result further confirms the underlining mechanism of the self-referencing detection scheme: binding to pathogen targets lead to detectable SERS signatures that differentiate dual SERS spectra from probe-alone spectra without separation of target-bound from unbound.

**Fig. 7:**
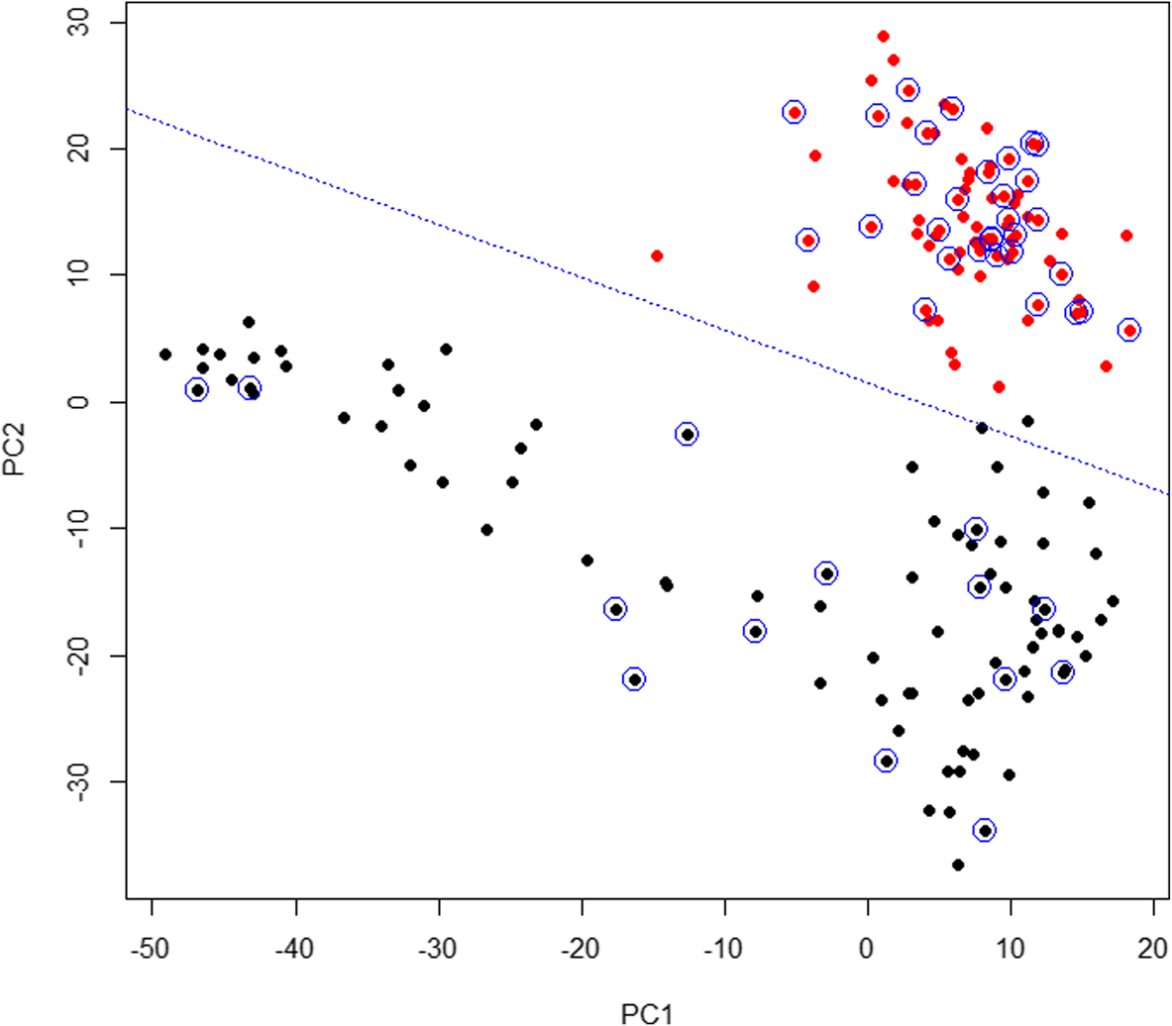
Classification of binding type and non-binding type spectra using SVM. Red dots represent binding type spectra; black dots represent non-binding type spectra. The dash line is the hyperplane showing the optimal linear separation. The blue circles indicate the support vectors

To further confirm the differentiation between positive signal (i.e., Dual spectra) and negative control (i.e., probe-alone spectra), a binary-based classification algorithm based on Support Vector Machine (SVM) was used in this study. Rather than performing prediction analysis using all of the spectroscopic information in the dataset, we use only those spectroscopic components with the strongest estimated correlation with bacterial target. After dimension reduction with PCA, we need to decide which principle components are important. The first 58 PCs, represented 80% of the total variance in the data set, were used for the following SVMs calculation. Linear kernel was used in our SVM model. The two types of SERS spectra (non-binding, probe alone and binding, dual) were collected from three separated batches of experiments. A sum of 166 spectra were used in the SVM testing, 79 were dual type; 87 were probe type SERS spectra. In this experiment the dataset is randomly split into two subsets: one containing 124 spectra was used to train the SVM model and the second dataset containing 42 spectra was used for the evaluation to validate the established SVM model by rotating the tested dataset into the same coordinate system as the training dataset. A shown in Fig. 7, the differentiation between the negative control and the positive I.D. is very good. The validation (Table 2) was evaluated by the accuracy percentage (>95%). It should be noted that here the negatively control was *E. coli* K12 alone (at 100 CFU/mL), and the positive sample was a mixture of *E. coli* K12 and *E. coli* O157:H7 (at 10:1 ratio), at 100 CFU/mL (with O157:H7 at 10 CFU/mL). All three nanoprobes were prepared to specifically bind to *E. coli* O157:H7 cells at three different epitopes. These results indicate that the self-referencing detection scheme allows the detection of a pathogen at very low level (10 CFU/mL) at the presence of 10 times higher non-target strains without any separation steps with very high fidelity, a specificity at sub-strain level.

**Table 2:** Result of the probe & dual spectra validation with a SVM model (the first 58 PCs are used only)

	SVM testing	
prediction	Probe	Dual
Probe	22	0
Dual	1	19

### Enrichment of samples by using Nano-DEP microfluidic device

Our results showed the LOD could be improved to 10^1^ CFU/mL by using the multiple epitopes self-referencing recognition strategy. Even though this SERS-based scheme could already provide such ultrasensitive and rapid detection results, the sensitivity needs further improvement for it to be a frontline solution to pathogen detection. It has been reported that the infectious dose of *E.coli O157:H7* bacteria is only 10 cells per gram of food and 0.2 CFU/mL in environmental sample, which underlines the desirability for extremely sensitive and specific pathogen detection [35].

**Fig. 8:**
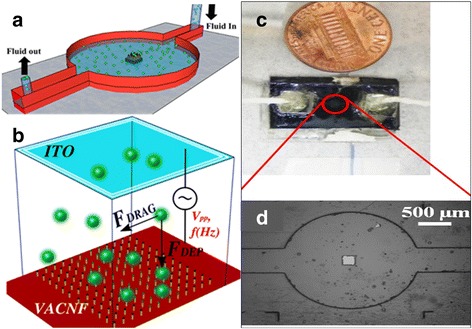
Schematic illustration of Nano-DEP microfluidic device. **a** the design of reservoir and fluid in/out channel; (**b**) carbon nanofiber nanoelectrode arrays (CNFNEAs) were embedded into this microfluidic device; (**c**) the actual size of this microfluidic device; (**d**) the dimension of the reservoir under microscope

In this study, we integrated a Nano-DEP microfluidic device into our detection platform. The nano-DEP device was used to enrich the samples before mixing with nanoprobes. Dieletrophoresis (DEP), a nondestructive electrokinetic transport mechanism, has been used to concentrate and separate various types of cells, especially microorganisms. DEP is the movement of particles due to polarization effects in non-uniform electric fields [36]. Nano-DEP utilizes carbon nanotube electrodes that generate DEP force that is one magnitude larger than that of normal DEP devices, hence the trapping/capturing efficiency is significantly improved [37]. This is critical when the sample to be concentrated is already a much diluted sample (e.g., 1–10 CFU/mL). Figure 8 illustrates the Nano-DEP microfluidic device used in this study for single cell trapping. In this lab-on-a-chip setup, the integrated vertical aligned carbon nanofiber nanoelectrode tip (in the square position, Fig. 8d) displayed an extremely high electric field gradient (10^20^ V^2^ m^−3^) when applied a voltage source, generating the so-called DEP force, a force exerted on a suspended dielectric particle (microbe) in the presence of a non-uniform electric field. The nano-DEP is strong enough to achieve cells trapping at high throughput by overcoming the hydrodynamic drag influence (Fig. 8b). The Nano-DEP device is considered act as a concentrator of target pathogen cells in this study. By passing through the bacteria-containing water samples (10^0^ CFU/mL or below) into the device for certain time periods when voltage turning on, the number of the cells trapped on the electrodes increase gradually. After certain duration of enrichment, the trapped target cells are released in one burst when the voltage is turned off, and they can be collected to a higher and detectable concentration level (10^1^ CFU/mL).

**Fig. 9:**
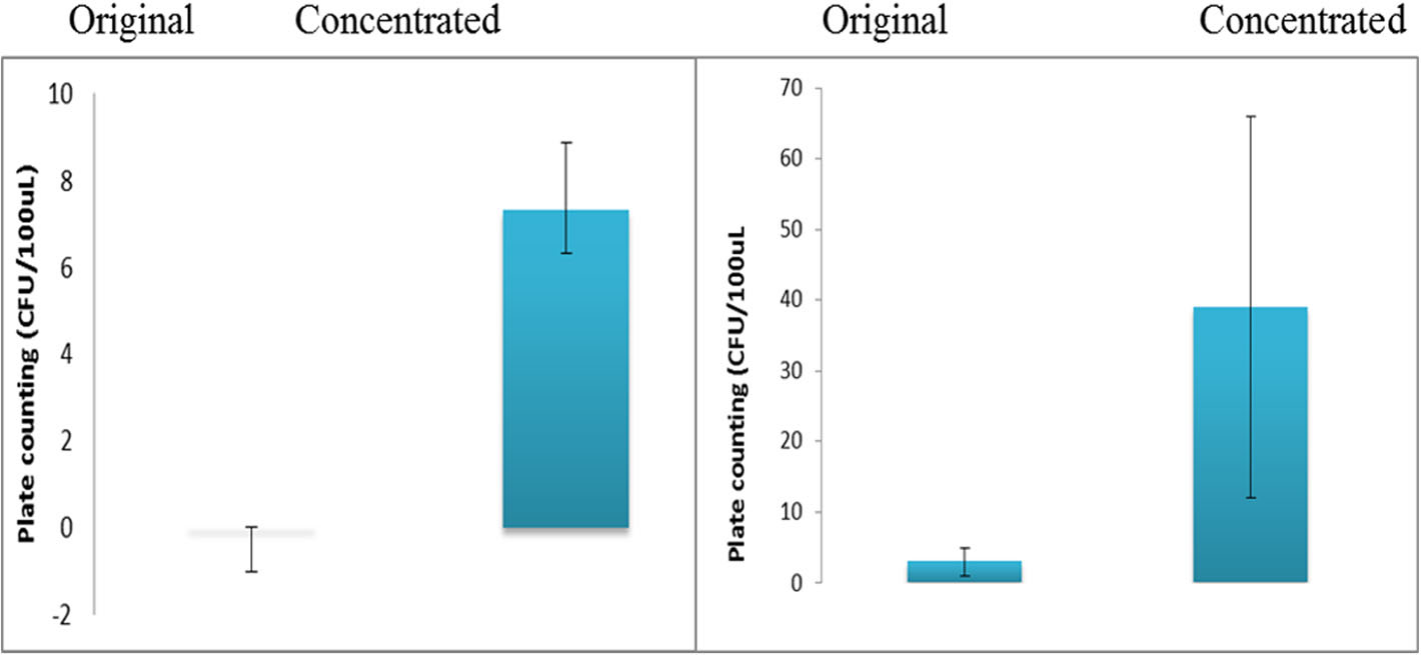
Plate counting results for two concentration ratios. Left: O:K=1:1 (CFU/mL in original solution); right: O:K=1:10 (CFU/mL in original solution)

The concentrated samples (volume from 1 mL to100 *μ*L; concentration from 10^0^ to 10^1^ CFU/mL) were collected by using microfluidic device. Two different mixed cell suspensions with different concentration ratios (between *E.coli O157:H7* and *E.coli K12,* namely O:K) were chosen in the testing. One was 1 CFU/mL (*E.coli O157: H7*): 1 CFU/mL (*E.coli K12*); the other was 1 CFU/mL (*E.coli O157:H7*): 10 CFU/mL (*E.coli K 12*). The concentration of original mixed cell suspension and the concentrated suspension were validated by plate counting. The results are shown in Fig. 9. From the results of both mixture tests, the 10 times enrichment efficiency was demonstrated. Since the multiplex self-referencing SERS nanoprobes can provide a high sensitive detection of the target bacteria, it is satisfied to obtain 10 times concentrating in the pre-enrich step using Nano-DEP.

## Conclusion

This novel multiplex self-referencing SERS pathogen detection scheme offered high sensitivity (10^1^ CFU/mL) and strain level discrimination by measuring the superimposed SERS signatures with multiple characteristic peaks. Furthermore, the superimposed spectra could be obtained directly with no washing being performed. Compared to the ELISA kits, this platform successfully isolated and identified bacteria in water samples without the need for repeated wash steps and secondary antibody reporting, hence significantly reducing the operation processes, detection time and the cost. In addition, this platform integrated with an excellent separation and concentration apparatus of Nano-DEP microfluidic device further improves the limit of detection (LOD) to 10^0^ CFU/mL. The integration of microfluidic devices with SERS detection yielded simple and miniaturized instrumentation that was suitable for the detection and characterization of small volume of chemical and biological analytes with high sensitivity and specificity. Multivariate statistical analysis techniques (PCA and SVM) firmly confirms the positive identification of targets in the presence of overwhelming non-target interference, with a detection accuracy above 95%. It has the potential to become a powerful, highly sensitive biosensor for onsite detection of pathogens at extremely low levels.
